# Selections and Abstracts

**Published:** 1897-07

**Authors:** 


### SELECTIONS AND ABSTRACTS.

### A Minister on "Faith-cure" and "Christian Science."

Rev. Walker Lewis, pastor of the First Methodist Church, Atlanta, does not believe in "Faith-cure" and "Christian Science." Of course he doesn't. He has just preached a strong and severe sermon, having these subjects for a theme, in the course of which he said in part:

The two forms of opposition to the gospel which I wish to speak of to-day are faith-cure and Christian science, falsely so called, each of them. The one is neither science nor Christianity; the other is neither cure nor faith. They are prodigious humbugs instead.

Faith-cure will take little of our time to-day. It is held by the advocates of this theory, that the practice and use of medicine is both unnecessary and sinful; that according to the Scriptures, disease may be cured by faith and prayer without the aid of physicians and prescriptions.

This is not the divine teaching. It is right and helpful to use prayer in the treatment of disease. But along with that, and going before it, natural means are to be used. First the natural, and then the spiritual. The annointing with oil, James advises, was advance practice in that day, and this was followed by prayer. The two were joined together. If medicine is useless, why was the earth made a vast pharmacy of "materia medica"? What God teaches in creation, he confirms in his own practice. He is, and he commands that he be approached as the Great Physician. "But did not Hezekiah rescue himself by prayer and faith"? These did much, but the fig poultice God prescribed did more to effect his healing. As between the two, the poultice was the better recourse; and you will show best sense, if your are sick, by sending first for a sensible physician, and then for your pastor, letting the first prescribe, and the other pray. Faith is helpful, but less so than a first-class watering place. Prayer is good for a man with a broken leg, but it is better to give him a plaster of Paris cast. Prayer for life won't lengthen it as surely as will a good beefsteak, well prepared and fully digested.

But faith-cure compared with Christian science is moderate madness, a little craze. According to this, both ministers and physicians are impostors, and they should be set aside. The church itself is a mistake, and is destined to fall. The issue between this humbug and the church is sharp. There is no common ground between them. It is an irrepressible conflict —one without armistice—and nobody can stand with Christian science without a denial of the faith once delivered unto the saints. It is downright apostasy from the Gospel!

Christian Science is spirit monism. That is, it teaches there is nothing but spirit and that what we call matter is only a semblance.

If there is no such thing as matter, then there can be no reality in sensation, and what we call pain and pleasure are alike illusive and baseless. It came to pass, therefore, that monism gave birth to pagan theosophy, sprung up in India before Christ, and devised to quiet the wretched and oppressed people with the doctrine that the most prosperous and the wretchedest were only apparently unlike; the one not suffering real pain, and the other not experiencing real pleasure. Pain and pleasure were alike as unreal as dreams. And so those vagarists have brought pagan philosophy into modern life, affirming we only think we suffer, but really do not. Mrs. Eddy says: "There is no imperfection; and what we call pain, sickness and death are mere errors of belief, and have no real being." Hear her on boils: "You say a boil is painful; but that is impossible, for matter without mind is not painful. The boil simply manifests your belief in pain, through inflammation and swelling, and you call this belief a boil." A boil, then, comes from bad belief. What a depraved believer Job must have been to have them from crown to toe. Pain is only a false belief; and the same is true of what we call pleasure. It is altogether unreal. Whenever you make a sane man believe that he is really as comfortable with the toothache as without it; whenever you can make a boy believe that he is having as good a time without dinner as his friend is having with mince pie and fruit cake; whenever you can persuade an African that there is no real pleasure in eating of an ice-cold Georgia watermelon when the mercury boils—then we may hear this doctrine with patience. Whoever teaches it or receives it is a fit subject for the asylum. Nobody of sound mind can believe such ravings. It is insanity.

---

### THE LOCAL TREATMENT OF CHRONIC GASTRIC CATARRH.

Local treatment may be applied in any stage of chronic gastric catarrh; but it must be varied somewhat in the different stages. The grade of inflammation, its character and persistence, likewise may require some modification of the treatment.

*First Stage.*—During the incipiency of chronic gastritis, local treatment is not so essential, except in bacterial cases, but is beneficial. It serves to modify the congestion when that is increased, and often allays dyspeptic symptoms, even when they are more marked than usual. The use of warm water (105 degrees), with bicarbonate of sodium (three per cent.), for washing out the stomach is frequently very valuable to remove the tenacious mucus usually adhering to the gastric mucous membrane, in this condition, and interferring with the proper mixing of peptic fluids with the food. The patient may drink a glassful of the solution before meals or it may be introduced into the stomach through the tube. If the tube is used, the stomach should be filled before allowing any reflow. The cold douche with water 80 to 60 degrees, is sometimes more grateful and helpful than the hot douche (110 to 125 degrees). A continuous effect may be secured by using a double tube and permitting the inflow and outflow to progress simultaneously; but care should be taken to keep the stomach distended sufficiently to have the solution come in contact with the entire gastric surface. The soda solution dissolves the mucus and the stream washes it away. Weak soapsuds may be used with the tube for the same purpose. More satisfactory in many instances is the use of a solution of Hydrozone. A glassful (fl. oz. viij) of a two or three per cent. solution may be given half an hour before meals. If used as a douche with the tube a five or six per cent. solution is not too strong, and two quarts the minimum amount. These douchings may be given one to six or seven times a week, according to the requirements of the case, and are frequently all the treatment this stage of chronic gastritis demands, except what changes are necessary in the diet.

*Second Stage.*—The inflammatory process is fully developed in the second stage, and while there may be weeks or months when there is little if any suffering, the treatment should be persistent. The cleansing of the gastric mucous membrane must be systematic and thorough. This is best accomplished with a solution of green soap or a five or eight per cent. solution of Hydrozone, introduced with the double tube. After first filling the stomach, inflowing and outflowing streams ought to remain about equal, or the outflow may exceed the inflow; the distension of the stomach may be maintained by retarding the reflow when necessary. This process can be beneficially accomplished by driving the solution into the stomach under increased air pressure; but when the proper apparatus for this method is not at hand the siphoning method with the single tube does very well. For home treatment, or when the tube cannot for any reason be used, a solution may be made for drinking. For this purpose a 2 or 3 per cent. solution of Hydrozone is prepared. The patient may take a glassful (8 oz.) half an hour before mealtime. He should lie down at once, remain five minutes on the back, then turn on the right side where he must remain during the remainder of the half hour. While the patient is on the back the solution comes in contact with every portion of the gastric mucous membrane, and turning to the right side facilitates the emptying of the stomach. By this process the offending mucus is dissolved and carried away and the organ is put into a proper condition to digest food. The use of Hydrozone has the additional advantage of checking the growth of the bacteria, and probably exhibits greater antiseptic properties than any other agent that can be used in the stomach with the same degree of safety. In obstinate cases this cleansing ought to precede every meal.

After the stomach is cleansed it should be treated with soothing, stimulating and healing applications. There are many preparations which can be so used,some of the best of which areglyceroleof bismuth and eucalyptol,the essential oils and Glycozone. Boric acid in 2 or 3 per cent. solution as a wash with the tube is sometimes very valuable. The other agents mentioned may be used with a nebulizer by means of which a vapor impregnated with the medicines can be passed into the stomach through a tube, the double tube being preferable. If it is not convenient to use a nebulizing apparatus, the glycerole mentioned, and especially Glycozone, may be administered by the mouth. In many cases, in fact, the latter mode of administering these agents is more desirable. These remedies encourage healing and materially enhance the patient's prospects of recovery. This is especially true in bacterial cases. When Hydrozone has been given before meals as already suggested for cleansing purposes, Glycozone may be administered in teaspoonful doses after meals with very satisfactory results. This line of treatment is frequently so successful that cases are temporarily relieved and possibly often a cure effected, particularly if the general treatment has been judiciously carried out.

If, for any reason, Glycozone cannot be employed the essential oils may be used. The oils of anise, peppermint, cubebs, and tar may be combined and used with a nebulizer as previously suggested. Although benefit may be derived from the administration of this combination, I prefer the Glycozone treatment. The use of hot water, 120 degrees or more, and the employment of cold water, 80 degrees to 40 degrees (F.), may give very happy results in certain severe cases.

*Third Stage.*—The condition referred to here is one of atrophy. The functions of absorption and motion may be fairly well performed. The chief difficulty then is with the digestion of proteids. The local treatment has two objects mainly, although a third is sometimes in mind. The first object is the removal of débris and foreign material. The second is the cleansing of the mucous membrane and the destruction of micro-organisms and their removal in order that the intestines may not receive bacterial products from the stomach. The third object sometimes kept in view in the local treatment by douching is a degree of stimulation of the functions of motion and absorption and the tonic effect to the gastric walls which follow these washings. The first object is accomplished by the use of sterilized water, or a 3 per cent. solution of sodium bicarbonate. Either tube may be used. The second object is effected by douching the walls with a green soap solution or a solution of Hydrozone. The latter agent in 5 per cent. solution as directed above, gives very pleasing results. The third object may be secured by using hot or cold water for the douche.—*Dr. J. M. G. Carter, American Therapist, Jan. 1897.*

---

### FERRATIN.

Ferratin was introduced by Prof. Schmiedeberg about four years ago. It was the result of scientific investigation, and full details were published in the *Archiv experimentelle Pathologique Pharmocolgie* and in other journals. Comparative and confirmatory investigations were made by Marfori and de Felippi; and all these reports were presented before the International Medical Congress at Rome.

Ferratin was adopted as a therapeutic agent, and is now in general use throughout Europe and in growing demand in the United States. The committee now revising the Russian Pharmacopœia for a fifth edition has recommended Ferratin for official adoption (see *Chemiker Zeitung* 31, '97.)

Ferratin has been tested physiologically and clinically by acknowledged authorities, and uniformly indorsed. The record is found in the Medical Annuals of 1894 to 1897, and in recent textbooks.

Two attempts to discredit the product were made about two years ago; one by a prejudiced critic, who set up a false standard and demolished it to his own satisfaction but without impressing others; and another by the originator of a competing therapeutic agent, whose specious arguments were promptly controverted by Prof. Marfori.

Recently an Italian, Cervello, has completed some experiments to show "absorption of iron from the alimentary canal," and an extract has been published in the *University Medical Magazine*, from which other journals will no doubt reprint it. Cervello claims to have demonstrated that "medicinal salts of iron form a soluble compound in the albumen in the intestine" and "the substance thus produced is strictly comparable to Schmiedeberg's and Marfori's ferratin, which is thus unnecessary, since the inorganic salts of iron are converted into it in the intestine."

Does a report ending in such a conclusion, worded to discredit Ferratin, deserve a serious answer? If it were not for the gratuitous and unjustified suggestion, that Ferratin "is thus unnecessary," the report would really be an indorsement of Ferratin.

How long does it take the "medicinal salts of iron" to change into Ferratin in the intestines? Does the transformation proceed under all circumstances? What percentage is finally made available and how much is assimilated?

Prof. Marfori has demonstrated (and his results have been verified by equally high authorities) that Ferratin contains 7 per cent. of iron, and that an average of 20 per cent. is absorbed. This is definite.

Cervello, moreover, has only rediscovered what Schmiedeberg and others who have made the experiments long ago stated, viz. (in the words of Prof. G. A. Fackler): "The physiological and therapeutic importance of Ferratin is based upon the fact that, after absorption, it is immediately available, while other compounds of iron, including the simple albuminates, must first be changed into Ferratin in order to become active agents."

Not so very long ago, the physician in prescribing for an anemic patient, directed that an iron nail be dropped into a bottle of red wine, and that patient drink a glassful three times a day. We have progressed rapidly and in quick stages since then. Ferratin is the ultimate point of this progress.

Physiological and clinical tests prove that Ferratin supplies the needed iron *to nourish the blood*—and hence *the system.*

On page 341 of Prof. Schmiedeberg's "Arznemitellehre" (latest edition) this eminent pharmacologist states: "The fact and effect of a craving for iron (Eisen-Hunger) can be experimentally proved on animals. A strong, frisky dog, after a moderate loss of blood, was fed for five months on pure milk only, and gradually became so weak that he refused further nourishment, became reduced in body-weight, tottered when on his legs, and finally was at the point of death. At this state 1 gram of Ferratin was added to the milk per day; the dog ate this with ravenous appetite, and within fourteen days had regained his weight and general condition to nearly equal the normal strength and activity possessed before commencement of the experiment."

Ferratin in eight-grain doses three times daily was recommended by Germain See, the late distinguished French therapeutist, for "those suffering from anemia from hard work, though apparently in good health; those, of both sexes, affected with chlorosis; those weakened by too rapid growth and puberty; those fatigued by study; and, in short, all in whom a diminution of red-blood corpuscles had ensued, due no matter to what cause."

---

### CONTINUED MALARIAL FEVER—A MEDICAL MYTH.

There has been more written and taught concerning continued malarial fever and typho-malarial fever than ever Dickens could collect concerning spontaneous combustion, and with as little reason. Any malarial remittent is continued if neglected, and any case of typhoid may have a malarial complication if malaria be present. But no malarial fever is necessarily a continued fever, no other poison being present, and any typhoid case can be rid of its possible malarial complication by a little specific treatment. The following facts speak strongly:

In the North where malaria is scarce in quantity and weak in quality, we never hear of continued or typho-malarial fevers. It is all typhoid there. In certain swamp regions of the South, where malaria runs riot, and is promptly treated, but where typhoid is unknown, we never hear of continued or typho-malarial fevers. The deduction is obvious, either the continued fever is a real case of typhoid or it is an improperly treated case of malarial remittent. Or else it is some other sort of a fever entirely different from either. The latter deduction is very improbable. Yet we hear of many cases of continued malarial or so called "slow fever"; eliminating typhoid, these cases are slow doctor. Experience has proven that every dose of malarial germs can be killed by an appropriate dose of quinine. But most physicians put their maximum dose of quinine at too small a figure. When the dose of germs in a particular case is too large for their dose of quinine they quit giving quinine and diagnosticate "slow fever." The maximum dose of quinine should not be a certain number of grains, but in a word—"*enough.*"

This was never more strikingly shown than in a late paper by Dr. Bedford Brown, of Virginia.*

He says: "Sixty grains of quinine per day of twenty-four hours, given in doses of ten grains every four hours, would invariably arrest the attack in seventy-two hours, and frequently in less time. Thirty grains per day would prolong the attack five or six days, especially if given in three grain doses every two hours. Twenty grains per day would prolong the attack between one and two weeks, and fifteen grains per day would prolong it from three to four weeks."

There you have a case of " slow fever." But the above was concerning malarial fever, probably of the ordinary remittent type. It is to be regretted that on the next page Dr. Brown does not advocate an even more vigorous treatment for the severer form, which

*See February, 1897, issue of this Journal, page 835.

even he calls the prolonged variety. He merely recommends twenty grains of quinine per day to partially control the symptoms, though he admits that thirty grains per day of the bisulphate will abort this "prolonged variety" in two weeks. Reasoning back on his own statements, sixty grains per day would abort it in one week, and one hundred and twenty grains per day would abort it inside the seventy-two hour limit. In actual practice it will take more in certan cases, as much as thirty grains in acid solution every four hours, or one hundred and eighty grains per day having been found necessary and satisfactory in one recorded case. If that much is needed, no bad effect need be feared. Generally the largest doses are required, because even the solution is but little absorbed in such cases. If but 25 per cent. is absorbed, of course the dose must be four times the dose really needed. Nothing that Dr. Brown said deserves more, or as much attention, and constant holding in memory every day, as his remarks on the hours to give quinine. More quinine is wasted by stringing out the doses into three-grain capsules than would supply the Cuban army. The pernicious custom is a surrender to the patient's whim; he must have capsules, and must have small capsules. If he will have capsules, use the ten-grain size. Ten grains every eight hours will do more to the germs than two grains every hour, and will not make the patient as nervous. It is doubtful wisdom to give more than three doses of quinine per day in any case, unless the absorption is manifestly deficient. Quinine is not eliminated in any great amount in eight hours. So no matter how large or small the daily dose, a better effect is to be expected if given but three times a day. No one, not even an infant, ever needs less than five grains at a single dose. For the absorption of quinine in an infant's stomach is so slight as to amount to no more than ten per cent. in some cases. Consequently to give less than five grains is to give none at all, especially if wrapped in a syrup and made more insoluble. The absorptive power of the stomach over quinine increases with age, apparently, in exact ratio to the increase in bodily weight. Hence it will be found that for successful practice in swamp regions, the dose of quinine for any ened between the age of one day and eight years is the same—viz., five grains.

Let the physician remember to give his quinine every eight hours, and let him remember that the dose is "enough," and "slow fever" will be a thing of the past. And let no man contradict this statement until he has faithfully tried the plan of treatment and failed. —*Charlotte Medical Journal.*

---

### HEREDITY, ITS SIGNIFICANCE IN LIFE INSURANCE.

The Omaho *Clinic* publishes an article on this subject by Dr. Rockwell, resident medical examiner for the Equitable Insurance Company, a part of which is as follows:

Understanding, then, that it is the tendency, and not the actual disease, that is transmitted, it is evident that unless there be an exciting cause the disease in question will not be developed in the offspring, and numerous instances of this are illustrated in families where some of the children acquire the disease of the parent, while others whose environments are different will escape entirely. A recognition of this point greatly simplifies the selection of those applicants for insurance in whose family record there is a blemish.

*Tuberculosis.*—Reliable statistics show that about twelve per cent. of all deaths are due to tuberculosis, and in selected risks the mortality is eight per cent.

Of all the exciting causes by far the most important is light weight. Thin people are very much more likely to develop tuberculosis than stout people, and this factor should enter largely into the consideration of risks in whose family there is a taint. In fact more importance should be placed on this point, considered in connection with the size of the chest, than on the family history. The general appearance of the applicant, whether he looks anti-consumptive or not, is likewise of great consequence.

The influence of a sedentary life, residence and occupation in crowded, ill-ventilated and dust-laden quarters, repeated attacks of acute bronchitis, excessive use of alcohol, and impaired nutrition from any cause, should be considered before deciding to accept or reject. There should be no strict iron-clad rules when dealing with these cases, for each one is entitled to be judged on its individual merits; but ordinarily the results will be better for the company if the applicant whose father or mother has died from consumption be postponed until he is thirty years of age.

When both parents have died he should have lived past the ages they attained, or at least until he is thirty-five. When one parent and one or more older brothers or sisters have died, the same rule should apply, and when both parents and one or more older brothers or sisters, he should be postponed until at least forty years of age.

*Insanity.*—According to Kirchhoff, thirty to forty per cent. of the cases exhibited an hereditary taint. Folsom believes that at least seventy-five per cent. can be made to show evidence of the trouble in the family history.

When it occurs in one parent only and the applicant is otherwise acceptable, we may disregard the flaw in the family record. Insanity in one parent and an uncle on the same side, or in a parent and a grandparent, predispose more strongly than even insanity in both parents. When more than one case occurs in the family record, and if the applicant has had a sunstroke, is using too much alcohol, has had syphilis, internal otitis, or nervous exhaustion, he should be declined. In considering the acceptance or rejection of these cases, close scrutiny should be made to discover, if possible, any tendency to ill health, physical deterioration, or loss of weight, for mental health, as a rule, depends on bodily health. When tuberculosis and insanity appear in the same family, the predisposition to the latter is greatly intensified. Severe mental strain, unpleasant domestic relations, intemperance and excesses of all kinds are important etiological factors and should be carefully regarded.

*Gout.*—Duckworth is a strong believer in the heredity of gout, and states that among the better classes, where more trustworthy and complete family histories can be obtained, the influence is shown in from fifty to seventy-five per cent. of the cases. In America these figures would probably be excessive.

When the disease appears in the family record, the subject, to be acceptable, should be entirely free from all evidence of the malady. If he is himself a sufferer he should be declined. When there is a strong family tendency to it the possible influence of exciting causes must be well considered; for example, the constant use of alcoholic beverages, overeating, especially of highly seasoned food, a sedentary life, proneness to fatty degeneration, and a vicious hygiene from any cause.

Endowment insurance may well cover many of these cases, for, unlike tuberculosis, the risk is greater after middle life than before.

Before issuing a policy to an applicant who gives a family history of gout, extraordinary care should be taken to investigate thoroughly the urine, heart, liver and digestive organs.

*Cancer.*—For some time past the impression has been growing in the minds of medical men that it will not be long before the specific germ of cancer will be discovered. That there is in some families a tendency to the affection cannot be denied, but it is no easy matter to state how far this influence extends to the offspring, and what chances the latter have of developing the disease. While Paget believes that one in three, Sibley one in nine, and Bryant one in ten, give evidence of an hereditary taint, the possibility of an infective source must be kept in mind and some of the cases be accounted for through the agency of similar environment. On the other hand, it is quite likely that certain peculiarities of the epithelial cells are transmitted, so that when sufficient external cause arises, these cells proliferate rapidly and go through the various stages of a malignant growth.

The family tendency to cancer should be very strong to justify us in declining an otherwise acceptable risk. Should more than two cases occur in the record, and the subject be over forty years of age, careful scrutiny should be made to determine the presence of a probable exciting cause; this being, in its widest sense, local irritation of any description. If found, the applicant should be kept under observation for a sufficient time to demonstrate the result.

Cases of diabetes, nephritis and heart disease may sometimes appear in sufficient number in the family record to justify declining the applicant on this ground alone. In these difficulties age is an important consideration, and by means of the endowment or other limited policies many of them can be profitably insured. The personal element here is a very strong factor, and not only the applicant's present physical condition should be carefully investigated, but his habits, occupation and mode of life call for close scrutiny, since oftentimes these influences are of paramount importance in deciding to accept or reject.

---

### Reciprocity in Medical Licensure.

Dr. William Warren Potter, of Buffalo, President of the National Confederation of State Medical Examining and Licensing Boards, chose this for the subject of his annual address at the seventh annual meeting of that body, held at Philadelphia, May 31, 1897.

*The Problem.*—The most important question now to be discussed pertains to the interstate exchange of licenses, and every friend of State control is interested in establishing this principle. It is one of the objects this confederation is laboring to accomplish, but a most difficult problem for solution. A national registration bureau is desirable, where legally qualified and reputable physicians may be recorded—physicians whose names appear on this register to be allowed to pass from State to State in the enjoyment of all privileges pertaining to the practice of medicine. Those chiefly agitating the question of reciprocity, however, are specialists who desire to spend profitable vacations at summer resorts, and do not relish the idea of taking State examinations in the localities chosen for their holiday practice. Another class of men, compelled by circumstances to change residence, is more deserving of sympathy; they take the examinations uncomplainingly. Shall a State require of its own citizens a complianee with its practice laws, while granting to thrifty summer specialists exemption from their operation? As the State laws forbid discrimination against the inhabitants of each, there is both a legal and a moral bar to such exemptions.

*Obstacles to Reciprocity.*—Equality of standards for admission to the study and practice of medicine is the only enduring basis on which reciprocity can be established. When the several States adopt a uniform level of preliminaries; a uniform period of collegiate training, including uniformity of methods of teaching; and finally, an absolute similarity in the methods of conducting State examinations and granting licenses, then reciprocity will be equitably and permanently established. It is important for the State medical examiners to come to an agreement on these several points, that they may act with intelligence on a common platform. The State imposes a postgraduate examination, and none should be admitted to it who are not holders of diplomas legally obtained from registered and recognized colleges. It is understood, of course, that there must be established a uniform system of recognizing and registering medical schools in the several States.

*The Solution—Legislative Enactments.*—The remedies lie in legislative enactments. Those who most loudly and persistently demand interstate indorsement aim their criticisms at examining boards; whereas, these have nothing to do with the question. The statutes in States that have established licensure prohibit interstate exchange, except between such as have equality of standards. The demands of the restless and migratory doctors must be taken to the State legislative halls. Meanwhile, the members of this confederation may assist in bringing the matter to a more speedy conclusion by acquainting their legislatures with the difficulties to be overcome, and by urgently recommending the adoption of such amendments to existing laws as will meet and remove the present defects. Great care must be exercised, however, in the preparation of amendments; the State laws are for the public weal, reciprocity is only for the few. Amendments to existing statutes should be proposed only through State medical examining boards, or State medical societies; they are familiar with the defects and best know the remedies needed. When legislatures can be persuaded to turn a deaf ear to all amendments that are proposed outside of official sources,it will be a happy day for the friends of State license. The object of this discussion is to divert further criticism of the delay of reciprocity into the proper channel. If legislators could be made to appreciate the fact that public health interests are involved in the question of State license; that every attempt to weaken the principle is a blow at public sanitation, and that higher standards of medical education mean better health for the people, then, perhaps, it would be easy to obtain and maintain the necessary laws to protect the commonwealths against that kind of ignorance, superstition, or super-refinement that always lurks in the environment of quackery.

